# Diagnostic accuracy of a three-protein signature in women with suspicious breast lesions: a multicenter prospective trial

**DOI:** 10.1186/s13058-023-01616-5

**Published:** 2023-02-14

**Authors:** Eun-Shin Lee, Yumi Kim, Hee-Chul Shin, Ki-Tae Hwang, Junwon Min, Min Kyoon Kim, SooKyung Ahn, So-Youn Jung, Hyukjai Shin, MinSung Chung, Tae-Kyung Yoo, Seungpil Jung, Sang Uk Woo, Ju-Yeon Kim, Dong-Young Noh, Hyeong-Gon Moon

**Affiliations:** 1 Division of Breast and Endocrine Surgery, Department of Surgery, Korea University Anam Hospital, Korea University College of Medicine, Seoul, Republic of Korea; 2 Division of Breast Surgery, Cha Gangnam Medical Center, CHA University School of Medicine, Seoul, Republic of Korea; 3 Department of Surgery, Seoul National University Bundang Hospital, Seongnam, Republic of Korea; 4 Department of Surgery, Seoul National University College of Medicine, Seoul Metropolitan Government Seoul National University Boramae Medical Center, Seoul, Republic of Korea; 5 Department of Surgery, Dankook University College of Medicine, Cheonan, Republic of Korea; 6 Department of Surgery, Chung-Ang University College of Medicine, Seoul, Republic of Korea; 7 Department of Surgery, Breast and Thyroid Center, Kangnam Sacred Heart Hospital, Hallym University, Seoul, Republic of Korea; 8 Center for Breast Cancer, National Cancer Center, Goyang, Republic of Korea; 9 Breast and Thyroid Care Center, Myongji Hospital, Goyang, Republic of Korea; 10 Department of Surgery, Hanyang University College of Medicine, Seoul, Republic of Korea; 11 Department of Surgery, Seoul St. Mary’s Hospital, College of Medicine, The Catholic University of Korea, Seoul, Republic of Korea; 12 Division of Breast and Endocrine Surgery, Department of Surgery, Korea University Hospital, Korea University College of Medicine, Seoul, Republic of Korea; 13 Department of Surgery, Korea University College of Medicine, Seoul, Republic of Korea; 14 Department of Surgery, Gyeongsang National University School of Medicine and Gyeongsang National University Hospital, Jinju, Republic of Korea; 15 Department of Surgery, Seoul National University College of Medicine, Seoul National University Hospital, Seoul, Republic of Korea; 16 Genomic Medicine Institute, Medical Research Center, Seoul National University, Seoul, Republic of Korea; 17 Cancer Research Institute, Seoul National University, Seoul, Republic of Korea

**Keywords:** Three-protein signature, Proteomic analysis, Breast cancer, Early detection, Prospective trial

## Abstract

**Background:**

Mammography screening has been proven to detect breast cancer at an early stage and reduce mortality; however, it has low accuracy in young women or women with dense breasts. Blood-based diagnostic tools may overcome the limitations of mammography. This study assessed the diagnostic performance of a three-protein signature in patients with suspicious breast lesions.

**Findings:**

This trial (MAST; KCT0004847) was a prospective multicenter observational trial. Three-protein signature values were obtained using serum and plasma from women with suspicious lesions for breast malignancy before tumor biopsy. Additionally, blood samples from women who underwent clear or benign mammography were collected for the assays. Among 642 participants, the sensitivity, specificity, and overall accuracy values of the three-protein signature were 74.4%, 66.9%, and 70.6%, respectively, and the concordance index was 0.698 (95% CI 0.656, 0.739). The diagnostic performance was not affected by the demographic features, clinicopathologic characteristics, and co-morbidities of the participants.

**Conclusions:**

The present trial showed an accuracy of 70.6% for the three-protein signature. Considering the value of blood-based biomarkers for the early detection of breast malignancies, further evaluation of this proteomic assay is warranted in larger, population-level trials.

This Multi-protein Assessment using Serum to deTermine breast lesion malignancy (MAST) was registered at the Clinical Research Information Service of Korea with the identification number of KCT0004847 (https://cris.nih.go.kr).

**Supplementary Information:**

The online version contains supplementary material available at 10.1186/s13058-023-01616-5.

## Introduction

Breast cancer incidence remains the highest in women worldwide and has substantially increased in Korea over the past several decades [1, 2]. Early detection is crucial to reducing mortality associated with breast cancer and morbidity during treatment [3]. Current breast cancer screening includes physical examination and mammography, although mammography has shown low accuracy in young women or women with dense breasts [4]. Mammography may also cause pain or discomfort during testing, and harm from irradiation may outweigh its benefits in young women [5, 6].

Blood-based markers can address the drawbacks of the current breast screening tools [7]. We recently reported a new proteomic assay that quantifies blood levels of three proteins (Mastocheck®) using multiple reaction monitoring mass spectrometry (MRM-MS) [8]. The three proteins used in the assay are apolipoprotein C-I (APOC1), carbonic anhydrase I (CA1), and neural cell adhesion molecule L1-like protein (CHL1). APOC1 was identified as a downregulated serum protein marker in an independent study [9]. Studies have suggested that the CA1 protein contributes to microcalcification in breast cancer and atherosclerosis [10, 11], and high levels of blood CA1 are detected in patients with breast cancer [10]. CHL1, a cell adhesion molecule, has been proposed as a tumor suppressor in breast cancer; however, its value as a diagnostic marker is unclear [12].

Three proteins were selected among the 124 proteins discovered in our previous proteomic experiments [13–16] based on the diagnostic performance of plasma levels of each protein in patients with breast cancer [8]. The three-protein signature was subsequently validated in a large-scale study using samples stored in biorepositories [17]. Additionally, this signature has been shown to increase the diagnostic accuracy of breast ultrasonography when used in combination [18]. Herein, we report the results of a prospective multicenter trial on the diagnostic accuracy of the three-protein signature in women with suspicious breast lesions.

## Methods

### Study design and participants

**Fig. 1:**
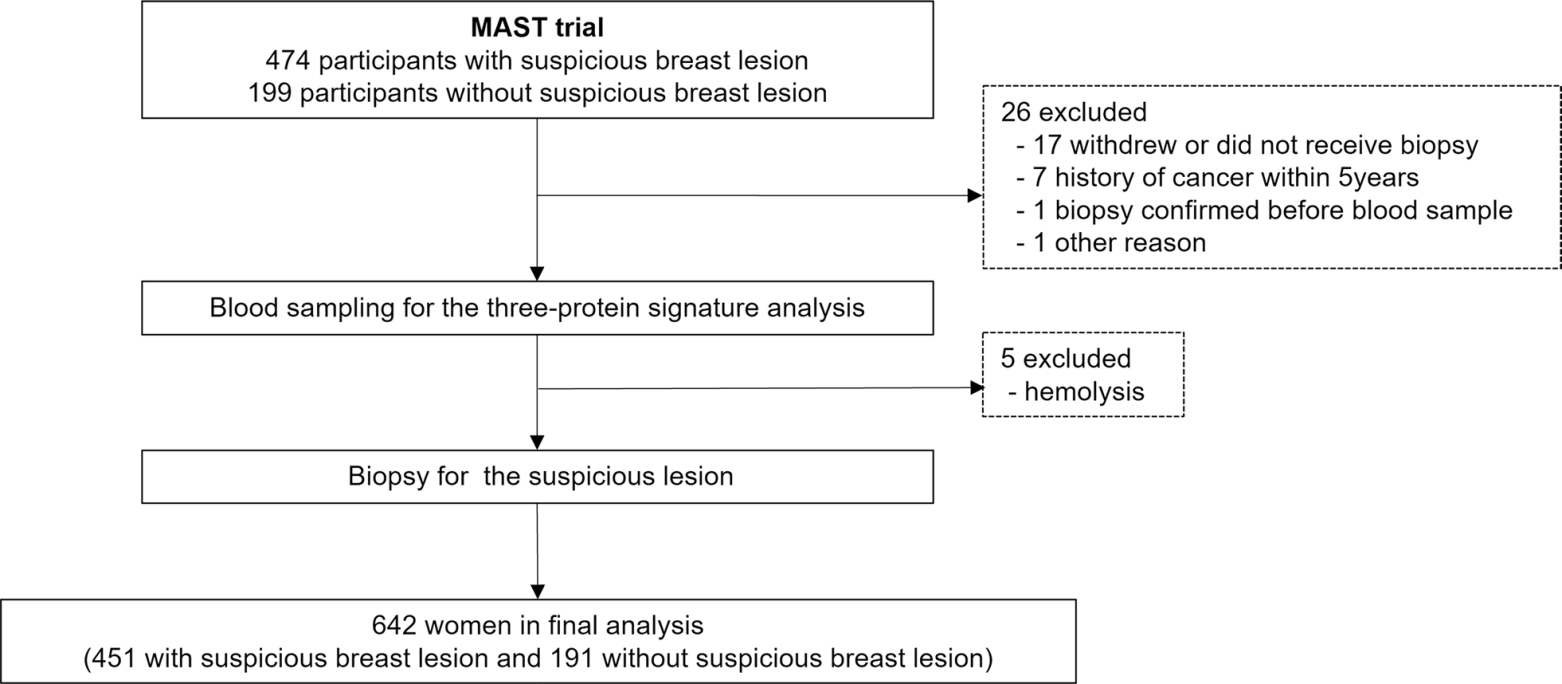
Study flow

This multi-protein assessment using serum to determine breast lesion malignancy (MAST) (KCT0004847) was a prospective multicenter trial conducted in 13 teaching hospitals in Korea. From August 2019 to September 2020, plasma and serum samples were collected from 471 participants with moderate to highly suspicious breast lesions identified by breast mammography or ultrasound, for which a breast biopsy was planned (Fig. 1). Moderate to highly suspicious breast lesions were defined as category 4B, 4C, or 5 of the Breast Imaging Reporting and Data System (BI-RADS) [19]. Women younger than 18 years of age, who had a personal history of breast cancer, or other malignancy within the five years were ineligible. Up to 20 ml of whole blood was drawn before the breast biopsy and was used for the three-protein signature. Demographic and pathological information was collected after the biopsy results were reported. A total of 451 patients were included in the final analysis after excluding patients for various reasons (Fig. 1). Additionally, the blood samples of 191 participants who showed no suspicious breast lesions (BI-RADS 1 or 2) were collected for the three-protein signature assessment. The three-protein signature (Mastocheck®, a regression model based on the relative expression of CHL1, APOC1, and CA1) was approved as an in vitro diagnostic tool using plasma samples by the Korean Ministry of Food and Drug Safety in Jan 2019. In this trial, serum samples were collected to determine the correlation between plasma and serum.

This trial was registered at the Clinical Research Information Service of Korea, a member of the World Health Organization (WHO) International Clinical Trials Registry Platform (ICTRP), with the identification number KCT0004847. The original version of the study protocol is provided in Additional file 1. The detail of sample processing and statistical analysis is described in Additional file 2: Additional Methods.

## Results

In this trial, 451 women with suspicious breast lesions and 191 with no suspicious lesions were included in the final analysis (Fig. 1). Among the 451 women with suspicious breast lesions, 313 were diagnosed with breast cancer through subsequent breast biopsies. Therefore, the analysis included plasma and serum samples from 313 patients with breast cancer and 329 women with biopsy-proven benign disease or no suspicious lesions in the breast. The clinical and demographic information of all participants is described in Additional file 3: Tables 1 and 2.

The sensitivity, specificity, and overall accuracy values of the three-protein signature for detecting breast malignancies in 642 women were 74.4%, 66.9%, and 70.6%, respectively (Table 1). The concordance index was 0.698 (95% CI 0.656, 0.739). The performance of the three-protein signature was not affected by the various clinical or demographic features of the participants and was not biased by site (*p* = 0.383) (Table 2). Notably, accuracy was not influenced by the mammographic density of the participants (*p* = 0.878). The three-protein signature assay was repeated using serum samples to determine the concordance rate with the results from the plasma samples. The results were concordant in 605 (94.2%) patients (Additional file 3: Table 1).

**Table 1:** Diagnostic accuracy of the three-protein signature

	All participants (*n* = 642)	Participants with suspicious breast lesion (*n* = 451)
Sensitivity (95% CI)	74.4% (69.6,79.3)	74.4% (69.6,79.3)
Specificity (95% CI)	66.9% (61.8,72.0)	60.9% (52.7,69.0)
Accuracy (95% CI)	70.6% (67.0,74.1)	70.3% (66.1,74.5)
Concordance index (95% CI)	0.698 (0.656, 0.739)	0.654 (0.596, 0.713)

**Table 2:** Distribution of demographic characteristics by whether the 3-protein signature is accurate

Variables		Number of women (%)		
		In Discordant group (n = 189)	In Concordant group (n = 453)	*p*-value
Age	Mean ± SD	52.1 ± 11.3	52.2 ± 11.4	0.586^1)^
Height (cm)	n	158 (83.6%)	391 (86.3%)	0.379^1)^
	Unknown	31 (16.4%)	62 (13.7%)	
	Mean ± SD	158.7 ± 5.6	158.2 ± 6.0	
Weight (kg)	n	158 (83.6%)	391 (86.3%)	0.867^1)^
	Unknown	31 (16.4%)	62 (13.7%)	
	Mean ± SD	59.1 ± 9.6	59.4 ± 9.5	
Anti-hypertensive medication	No	156 (82.5%)	374 (82.6%)	0.995^2)^
	Yes	33 (17.5%)	79 (17.4%)	
Dyslipidemia medication	No	164 (86.8%)	396 (87.4%)	0.824^2)^
	Yes	25 (13.2%)	57 (12.6%)	
Diabetes medication	No	172 (91.0%)	423 (93.4%)	0.293^2)^
	Yes	17 (9.0%)	30 (6.6%)	
Synthroid medication	No	177 (93.7%)	423 (93.4%)	0.898^2)^
	Yes	12 (6.4%)	30 (6.6%)	
Other medication	No	160 (84.7%)	391 (86.3%)	0.583^2)^
	Yes	29 (15.3%)	62 (13.7%)	
Breast density grade in mammogram^a^	n	84 (44.4%)	185 (28.8%)	0.878^3)^
	Unknown	105 (55.6%)	268 (41.7%)	
	Grade 1 (Almost entirely fatty)	3 (3.6%)	4 (2.2%)	
	Grade 2 (Scattered fibroglandular densities)	11 (13.1%)	24 (13.0%)	
	Grade 3 (Heterogeneously dense)	42 (50.0%)	98 (53.0%)	
	Grade 4 (Extremely dense)	28 (33.3%)	59 (31.9%)	
By site	13 institutions	189 (100%)	453 (100%)	0.383^2)^

SD; standard deviation

^a^American College of Radiology (ACR) BI-RADS Breast Density Categories

^1)^Wilcoxon rank sum test ^2)^Chi-square test ^3)^Fisher exact test

**Fig. 2:**
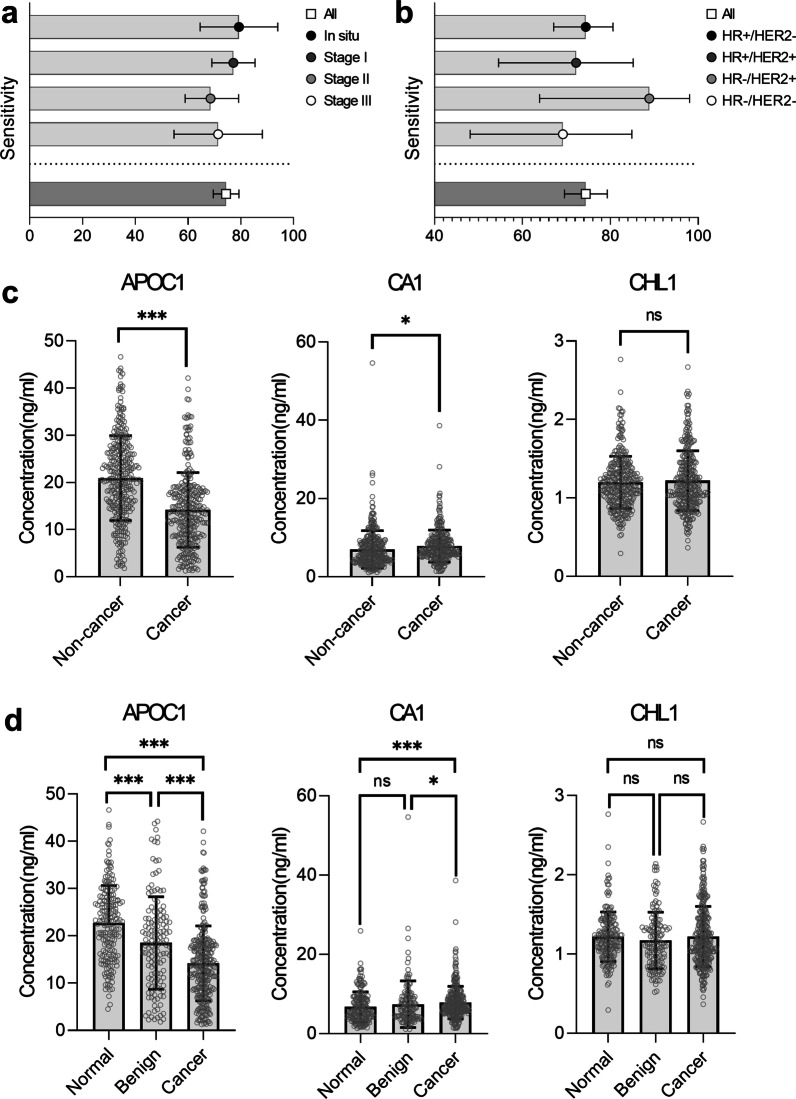
Results of the three-protein signature analysis. **a**, **b** depict the sensitivity of the three-protein signature in different cancer stages and subtypes, respectively. The sensitivity of the three-protein signature showed no statistical differences across AJCC stages and molecular subtypes (*p* = 0.859 and *p* = 0.902, respectively). **c** The concentration of each protein in all participants. APOC1 and CA1 levels were statistically different between 313 patients with breast cancer (Cancer group) and 329 participants (Non-cancer group) with no suspicious breast lesions (Normal group) or biopsy-proven benign breast lesions (Benign group), whereas CHL1 did not show such a difference. Wilcoxon rank sum test was used to compare differences between two groups because the concentration of each protein was not normally distributed. **d** Depict the concentrations of the three markers in three participants’ group. APOC1 were significantly different between Normal group, Benign group, and Cancer group. While CA1 levels of Cancer group were higher than those of Normal group and Benign group, CA1 did not show significant difference between Normal group and Benign group. *p*-values calculated using Dunn’s nonparametric comparison for post hoc Kruskal–Wallis testing. *p < 0.05, **p < 0.01, ***p < 0.001 and ns non-significant

Although the three-protein signature showed trends for higher sensitivity for in situ tumors and the American Joint Committee on Cancer (AJCC) stage I cancers, the sensitivity of the three-protein signature did not show statistically significant differences across AJCC stages (*p* = 0.859, Fig. 2a). Additionally, breast cancer subtypes, as defined by hormone receptor status and human epidermal growth factor receptor 2 (HER2) expression, did not show statistically significant association with the sensitivity of the three-protein signature (*p* = 0.902, Fig. 2b). Figure 2c shows the levels of the three protein markers among all participants. The levels of APOC1 and CA1 showed statistically significant differences between 313 patients with breast cancer and 329 participants with BI-RADS C1 or 2 breast imaging or biopsy-proven benign breast lesions. However, the CHL1 did not show such differences in the present study. The levels of APOC1 showed statistically significant differences between participants who had no suspicious lesion, patients who had biopsy-proven benign breast lesions, and patients with malignant breast lesions (*p* < 0.001, Fig. 2d). While the CA1 levels of patients with breast malignancy were significantly higher when compared to those of participants with biopsy-proven benign lesions or participants with no suspicious lesions (*p* = 0.019 and *p* < 0.001, respectively), the levels of CA1 did not show significant difference between the latter two groups (*p* = 0.482) (Fig. 2d).

## Discussion

To our knowledge, this is the first report of a prospective multicenter trial that addressed the accuracy of blood-based proteomic diagnostic markers for breast cancer. Although many studies have focused on discovering blood-based protein biomarkers for the early detection of breast cancer, the clinical value of these biomarkers has not yet been tested in a prospective clinical trial setting [20]. Our results demonstrated that the sensitivity of the three-protein signature was 74.4%, which is comparable with the results of our previous reports using archived blood samples [17]. Interestingly, the sensitivity of the three-protein signature was not affected by the mammographic density of participants or other clinicopathological factors. As low sensitivity has been a major hurdle for using traditional serum markers of breast cancer in the clinic [21], the present findings suggest that this proteomic-based assay may be a useful tool for breast cancer diagnosis in women with dense breasts. Indeed, our previous study using large-scale biorepository samples suggested that combining the three-protein signature with mammography can improve diagnostic accuracy in women with dense breasts [22].

Our study carries several limitations. First, no follow-up data were available for the 191 participants who showed no suspicious lesions in breast imaging. Second, detailed pathologic information on the 61 (19.5%) participants with breast cancer was not available. While the stage or subtype did not influence the accuracy of the three-protein signature in the remaining 252 patients, it would be important to prospectively address this issue in a larger prospective cohort since the current trial was not designed for determination of the stage- or subtype-specific accuracy of the assay. Third, unlike APOC1 and CA1, which showed significant differences between benign and cancer patients, CHL1 levels failed to show such differences in the current trial. Further efforts to discover additional biomarker candidates are warranted to improve the diagnostic accuracy of the assay. Finally, the demographic features and co-morbidities showed differences between patients with breast malignancy and participants without suspicious lesions or with benign breast lesions (Additional file 3: Table 2). This finding also suggest that it require further data form a large cohort to verify the value of three-protein signature.

Screening tests are subject to high specificity to ensure a low rate of false positives, minimizing unnecessary diagnostic workups. The results of this trial revealed that the three-protein signature showed a moderate degree of specificity (60.9 (52.7–69.0)%) for 451 women with suspicious lesions and all participants (66.9 (61.8–72.0)%). A larger clinical trial with asymptomatic women undergoing breast cancer screening is required to determine the recall rates of the three-protein signature. Considering the benefits of blood-based biomarkers in breast cancer screening [7], our data warrant further efforts to validate the value of this proteomic assay. This prospective study showed that a three-protein signature based on MRM-MS is a sensitive tool for breast cancer diagnosis. A future large clinical trial is warranted to determine its value in breast cancer screening.

## Supplementary Information

**Additional file 1**. MAST trial protocol.

**Additional file 2**. Additional Methods.

**Additional file 3**. Additional Table 1 and Additional Table 2.

## Data Availability

All clinical data generated or analyzed during this study are summarized in this published article and its Additional files. The proteomic data that support the findings of this study are available from the corresponding author on reasonable request.

## Abbreviations

MAST: Multi-protein assessment using serum to determine breast lesion malignancy
MRM-MS: Multiple reaction monitoring mass spectrometry
APOC1: Apolipoprotein C-I
CA1: Carbonic anhydrase I
CHL1: Neural cell adhesion molecule L1-like protein
BI-RADS: Breast imaging reporting and data system
WHO: World Health Organization
ICTRP: International clinical trials registry platform
AJCC: American Joint Committee on Cancer
HER2: Human epidermal growth factor receptor 2

## Declarations

### List of approval number at each site

Seoul National University Hospital (1905-175-1036), Seoul National University Bundang Hospital (E-1909-564-406), Seoul Metropolitan Government Seoul National University Boramae Medical Center (20-2019-61), Dankook University Hospital (2019-09-001), Chung-Ang University Hospital (1982-004-384), Hallym University Kangnam Sacred Heart Hospital (2019-08-009), National Cancer Center (NCC2019-0298), Myongji Hospital (2019-09-010), Hanyang University Hospital (2019-09-011), Catholic University of Korea Seoul St. Mary's Hospital, College of Medicine (KC19TDDI0796), Korea University Anam Hospital (2020AN0088), Korea University Guro Hospital (2020GR0119) and Gyeongsang National University Hospital (2020-01-030).
