# Supplementary material for: Diagnostic accuracy of a three-protein signature in women with suspicious breast lesions: a multicenter prospective trial
Source: Breast Cancer Res. 2023 Feb 14;25:20. doi: 10.1186/s13058-023-01616-5 (PMC9930228; doi:10.1186/s13058-023-01616-5)
Supplement: Supplementary file 1 — Additional file 1. MAST trial protocol. [file 13058_2023_1616_MOESM1_ESM.pdf]

# Study Protocol

## Multi-protein Assessment using Serum to determine the breast lesion malignancy: a prospective multicenter Trial; (MAST)

| | |
|---|---|
| **Protocol Number** | MAST |
| **Product** | MASTOCHECK |
| **Study Phase** | Post-approval |
| **Regulatory Agency Identifier Number(s)** | N/A |
| **Sponsor** | Bertis Inc.<br>614, Nonhyeon-ro, Gangnam-gu, Seoul, 06108,<br>Replublic of Korea |
| **Version Number and Date** | V1.6 *2021/Sep/01* |

**CONFIDENTIAL**
The information contained in this document, especially any unpublished data, is the property of Bertis Inc. and therefore is provided to you in confidence as an investigator, potential investigator, or consultant, for review by you, your staff, and an applicable Ethics Committee or Institutional Review Board. It is understood that this information will not be disclosed to others without written authorization from Bertis Inc. except to the extent necessary to obtain informed consent from persons to whom the drug may be administered.

# Protocol Synopsis

| | |
|---|---|
| **Sponsor** | Bertis Inc. |
| **Product** | MASTOCHECK |

| | |
|---|---|
| **Full Study Title** | Multi-protein Assessment using Serum to determine the breast lesion malignancy: a prospective multicenter Trial |
| **Protocol Number** | MAST |
| **Classification/Phase** | Post-approval |
| **Indication for Use** | Breast Cancer |
| **Investigational Device** | MASTOCHECK in vitro diagnostic software |
| **Objective** | Conduct a prospective clinical trial in patient and healthy control groups for evaluation of a multi-marker algorithm and decision cutoff value derived from early breast cancer biomarkers (Carbonic anhydrase 1, Apolipoprotein C-1, Neural cell adhesion molecule L1-like protein) identified with proteomics-based technology.<br>And discover additional biomarkers to improve diagnostic ability.<br><br>Primary endpoints:<br>1. Sensitivity<br>2. Specificity<br>3. Receiver operating characteristic (ROC) curve (concordance index)<br><br>Secondary endpoints:<br>1. Positive Predictive Value<br>2. Negative Predictive Value<br>3. Accuracy |
| **Study Population** | 1. Patients with suspected cancer on diagnostic imaging; BI-RADS category ≥ C4b<br>2. Patients without suspected cancer (healthy control) on diagnostic imaging; BI-RADS category C1-2<br><br>Target biomarkers for analysis:<br>- Carbonic anhydrase 1 (CAH1)<br>- Apolipoprotein C-1 (APOC1)<br>- Neural cell adhesion molecule L1-like protein (NCHL1) |
| **Inclusion Criteria** | Cancer patient group:<br>1) Patients who have a lesion of BI-RADS category of ≥C4b through breast cancer imaging (mammography, ultrasound).<br>2) Women over 18 years old |

| **Sponsor** | Bertis Inc. |
|---|---|
| **Product** | MASTOCHECK |

| | |
|---|---|
| | 3) Patients with no history of other cancer diagnoses, including previous breast cancer<br>4) Patients voluntarily participating in this clinical study with an informed consent<br><br>Healthy control group:<br>1) Patients in BI-RADS category C1-2<br>2) Women over 18 years old<br>3) Patients with no history of other cancer diagnoses, including previous breast cancer<br>4) Patients voluntarily participating in this clinical study with an informed consent |
| **Exclusion Criteria** | Common criteria<br>1) Patients with recurrent breast cancer<br>2) Patients diagnosed with cancer in other organs within 5 years<br>3) Patients with other cancers confirmed by metastasis test<br>4) Systemic condition that makes it difficult to understand and submit informed consent |
| **Number of Subjects** | A total of 677 subjects<br>- 477 patients with suspected cancer; BI-RADS category ≥C4b<br>- 200 healthy control subjects; C1/2<br><br>*No vulnerable subject included |
| **Duration of Subject Participation** | Prospective Blood Collection:<br>Study participants will attend the following visits:<br>Visit 1: Informed consent, eligibility criteria evaluation, blood collection<br>- If the subject had an imaging before study screening, information can be collected from the previous visit. |
| **Number of Sites** | 1 |
| **Planned Study Duration** | 19 JUL 19 – 30 JUN 21 |
| **Statistical Considerations** | A prospective clinical trial is to be conducted on undiagnosed patients to confirm the diagnostic capabilities of the developed algorithm.<br>After measuring the concentration of three proteins in plasma (carbonic anhydrase 1, neural cell adhesion molecule L1-like protein, apolipoprotein C-1), an evaluation will be conducted by calculating the following: |

| | |
|---|---|
| **Sponsor** | Bertis Inc. |
| **Product** | MASTOCHECK |

| | |
|---|---|
| | sensitivity, specificity, positive predictive value, negative predictive value, accuracy, and likelihood ratio.<br>Effectiveness evaluation can be conducted by comparing the results of protein quantification analysis between cancer group and non-cancer group with diagnosis confirmed by tissue biopsy |
| **Expected Outcome** | The results of previous studies showed high levels of CAH1 and NCHL1 and low levels of APOC1 in breast cancer patients' blood. If the markers show significant changes in accordance with changes in tumor burden (before/after surgery, or before/after neoadjuvant chemotherapy), the markers may serve as a tool for early diagnosis of breast cancer. |

## Schedule of Activities (SoA)

| Procedure | Visit 1 | Notes |
|---|---|---|
| Obtain consent | V | - |
| Inclusion/exclusion criteria review | V | - |
| Basic information survey* | V | - |
| Medical history* | V | - |
| Physical examination* | V | - |
| Blood test | V | - |
| Imaging examination (mammography, breast ultrasound)* | V | - |
| Biopsy | V | - |

*If the subject had an imaging before study screening, information can be collected from the previous visit.

## Table of Contents

Protocol Synopsis.....2
Schedule of Activities (SoA).....5

**Table of Contents.....6**

**1. Introduction.....8**
1.1. Background.....8
1.2. Benefit/Risk Assessment.....12

**2. Study Objectives, Endpoints.....13**
2.1. Study Objectives.....13
2.2. Study Endpoints.....13

**3. Stakeholder Information.....14**
3.1. Name and address of coordinating institution.....14
3.2. Name and position of coordinating investigator and co-investigators.....14
3.2.1. Coordinating investigator.....14
3.2.2. Co-investigators.....14
3.2.3. Main contact at coordinating institution.....14
3.2.4. Investigational device manager.....15
3.3. Research sponsor name and address.....15
3.3.1. Research sponsor name and address.....15
3.3.2. Monitor name and address.....15
3.4. Research funding party name and address.....15

**4. Study Population.....16**
4.1. Inclusion Criteria.....16
4.1.1. Test Group (breast cancer suspected).....16
4.1.2. Control Group (healthy expected).....16
4.2. Exclusion Criteria.....16
4.3. Participant Discontinuation/Withdrawal from the Study.....16

**5. Study Intervention(s) and Concomitant Therapy.....17**
5.1. Dosage and Administration.....17
5.2. Concomitant Therapy.....17
5.3. Discontinuation of Study Intervention.....17

**6. Study Assessments and Procedures.....18**
6.1. Study Assessment.....18
6.1.1. Observation items.....18
6.1.2. Assessment items.....18
6.2. Sample Handling Procedures.....18
6.2.1. Sample collection.....18
6.2.2. Plasma and serum separation condition.....18
6.2.3. Sample preparation.....18
6.2.4. Target marker concentration analysis.....19

6.2.5. Statistical algorithm analysis ....................19
6.2.6. Proteomic profiling ....................19

**7. Data Analysis ....................20**
7.1. Medical Device Code Name ....................20
7.2. Data Analysis Procedure ....................20

**8. Statistical Considerations ....................21**
8.1. Sample Size Calculation ....................21
8.2. Randomization method ....................22
8.3. Interim Analysis ....................22

**9. Safety and Adverse Events ....................23**

**10. Supporting Documentation and Operational Considerations ....................24**
10.1. Regulatory and Ethical Considerations ....................24
10.2. Informed Consent Process ....................24
10.3. Compensation plan for study subject ....................24
10.4. Recruitment strategy ....................25
10.5. Personal information protection plan of study subjects ....................25
10.6. How to store and dispose of human materials ....................25

**11. References ....................28**

# 1. Introduction

## 1.1. Background

Currently, various factors such as female sex hormones, family history, past medical history, fertility history, and dietary habits are being discussed as the cause of breast cancer, but solid correlations with any of the aforementioned factors are yet to be identified. The incidence of breast cancer in Korean women continues to increase, and the increase in the incidence of breast cancer and the mortality rate from breast cancer is expected to continue for a considerable period in the future, as the westernization of Korean society continues. Breast cancer usually causes symptoms such as invasion of surrounding tissues or metastasis to lymph nodes due to the growth of cancer cells. However, most cases can be detected with self-examination without any symptoms. Thus, it is very important to effectively diagnose breast cancer early in order to reduce the mortality rate from breast cancer (1).

While various methods are used in combination to diagnose breast cancer, 70% of breast cancer patients are visiting the hospital after an abnormal finding in self-examination. However, it is very difficult to distinguish between malignant tumor and benign cyst through self-examination. Other breast cancer diagnosis methods include X- ray mammography, ultrasonography, fine-needle aspiration biopsy (FNAB), and magnetic resonance imaging (MRI), but it is important to ultimately confirm the diagnosis through a tissue biopsy.

X-ray mammography is a method of examining breasts with X-ray imaging, which is excellent for discriminating whether the lump is benign or malignant, but it is also the most effective method for identifying early breast cancers with hidden lumps that are too small to be identified with self-examination. However, mammography has a disadvantage in that the diagnosis rate is low in populations such as young women, who typically have a high density of mammary glands, or Korean women, who typically have small and fibrous breasts. There are also concerns that frequent radiological imaging may induce breast cancer. As an alternative to mammography, ultrasonography is widely used. Although the ultrasonography is effective in distinguishing between cysts and hard lumps, the ability to discriminate between malignant tumors and benign cysts is poor.

As a method to compensate for the shortcomings of these existing diagnostic methods, there have been attempts to diagnose breast cancer by measuring the concentration of tumor markers in the patient's plasma (2).

Although the value of these tumor markers as diagnostic or prognostic factors is being studied, the use of such markers is limited at present and there are no breast cancer markers that have official recommendations for inclusion into standard diagnostic protocols. Therefore, the researchers at this investigational site conducted five studies using tissue, blood, and cell samples, and selected 124 proteins as the primary candidates for breast cancer biomarkers.

The first study was a tissue-based experiment. A 2-dimensional differential gel electrophoresis (2-D DIGE) was conducted on 3 breast cancer tumor samples and 3 non-tumor samples. The 53 proteins that showed ± 1.8 fold difference (P-value <0.05) between the two groups were selected as candidates (3).

In the second study, ICAT (Isotope coded affinity tag) was conducted using the blood of 6 breast cancer patients and the blood of 6 normal subjects, and 33 proteins with a difference of more than 1.5 fold (P-value <0.05) between the two groups were selected (4).

The third study was conducted using mTRAQ® with blood from 6 breast cancer patients and blood from 6 normal subjects, and 8 proteins with a difference of more than 2 fold (P-value <0.05) between the two groups were selected as candidates for breast cancer biomarkers (5).

In the fourth study, 2-dimensional electrophoresis (2-DE) was performed using cells. 8 proteins with a difference of more than 2 fold were selected as candidates (16). In the fifth study, 29 proteins with proteins with a difference of more than 1.5 fold between breast cancer and normal groups were selected from various literatures (6-8).

A total of 124 proteins were selected as primary protein candidates excluding 7 overlapping proteins among the 131 breast cancer biomarkers selected as described above (9).

Among the 124 primary protein candidates, 56 proteins that can be analyzed by Multiple Reaction Monitoring (MRM) in blood were selected as the secondary candidates (10). For the 56 proteins, plasma samples of 80 breast cancer patients who show breast cancer stage distribution as shown in Table 1 and plasma samples of 80 normal patients were verified using Multiple Reaction Monitoring (MRM) analysis.

▶ Mechanism of Multiple Reaction Monitoring (MRM)

(1) The precursor ion to be analyzed is preferentially selected from the first mass filter, Q1,
(2) and the selected peptide from Q2, which acts as a collision cell, is fragmented.
(3) A specific ion can be monitored because the fragmented product ions are filtered again at Q3, the third quadrupole.

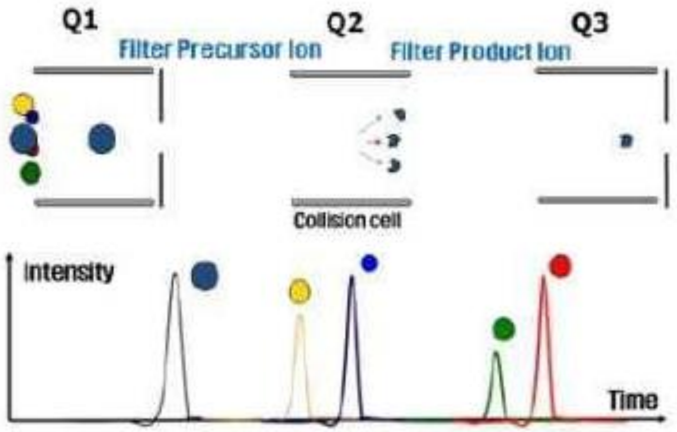


**Figure 1. Mechanism of Multiple Reaction Monitoring (MRM)**

As a result, 11 out of 56 proteins showed significant difference in protein concentration between the normal group and breast cancer patient group with a P-value of <0.05. In particular, 3 proteins (carbonic anhydrase 1; CAH 1, neural cell adhesion molecule L1-like protein; NCHL1, apolipoprotein C-1; APOC1) showed a P-value of <0.001. The researchers determined the 3 proteins would be useful for breast cancer prediction and diagnosis and the proteins were finally selected as breast cancer biomarkers (11).

The final 3 proteins selected showed a P-value of <0.001 in all stages of breast cancer, including early stages (stage 1 and 2) as shown in Figure 1. The specificity for all breast cancer stages including early stages (stage 1 and 2) was the same as 78.75%, and the sensitivity was 78.75% for all stages and 80.64% for early stages (stage 1 and 2), respectively.

Therefore, the researchers at this investigational site utilized the 3 proteins (carbonic anhydrase 1; CAH 1, neural cell adhesion molecule L1-like protein; NCHL1, apolipoprotein C-1; APOC1) that were finally selected through verification, and established the following primary candidate formula for the early breast cancer screening model [Result = (0.604 X CAH1 concentration) + (7.7575 X NCHL1 concentration) - (0.523 X APOC1 concentration)] using logistic regression analysis (11).

In addition, the primary candidate formula established through verification was validated for the finally selected 3 proteins, using plasma samples from new donors whose samples were not included in the verification process (99 breast cancer patients and 99 normal subjects). As a result, as shown in Figure 1, sensitivity was 69.70% and specificity was 70.10% in all stages, while sensitivity was 77.21% and specificity was 68.13% in early breast cancer (stage 1 and 2) (11).

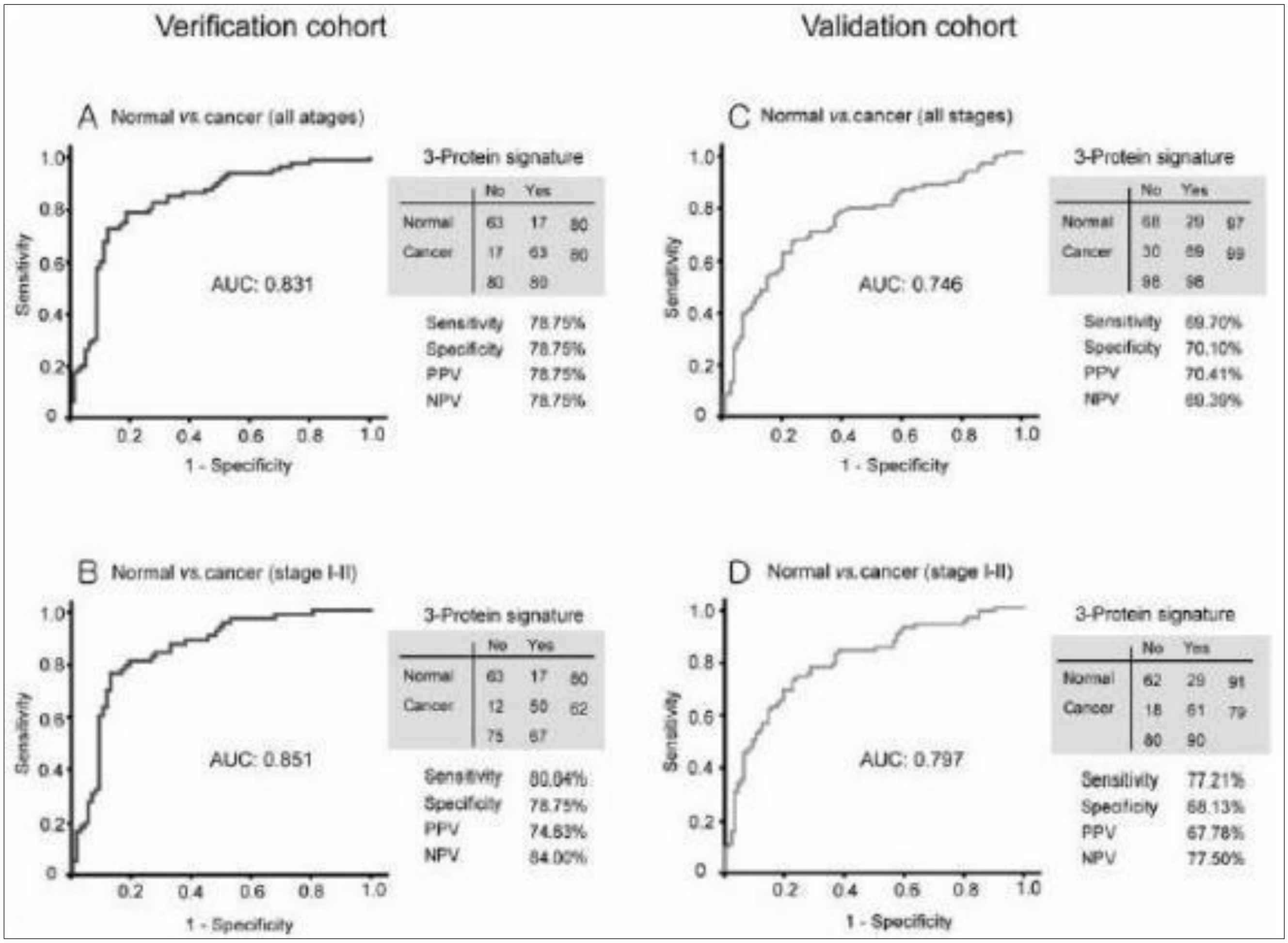


**Figure 2. Verification and validation result of 3-proteins selected for breast cancer biomarkers**

For reference, it is known from the literature that the 3 proteins selected as early breast cancer biomarkers have tendencies as shown in Table 1 and have functions as shown in Table 2.

| Protein name | Breast cancer | Lung cancer | Stomach cancer | Colorectal cancer | Pancreatic cancer |
|---|---|---|---|---|---|
| CAH1 | Concentration increase | | | Concentration decrease | |
| NCHL1 | Concentration increase | Concentration decrease | Concentration decrease | Concentration decrease | Concentration increase |
| APOC1 | Concentration decrease | | | | |

**Table 1. The propensity of the 3 proteins selected for early breast cancer biomarkers**

| Protein name | Function |
|---|---|
| APOC1 | It acts as an inhibitor of lipoproteins that bind to low-density lipoprotein (LDL) receptors, LDL receptor-related proteins, and very low-density lipoprotein (VLDL) receptors. Combined with high-density lipoprotein (HDL) and triacylglycerol-rich lipoproteins in plasma, it accounts for about 10% of the protein in VLDL and about 2% of the protein in HDL. It directly interferes with fatty acid intake and is also a major plasma inhibitor of cholesterol ester transfer protein (CETP). It binds free fatty acids, reduces intracellular esterification, regulates the interaction of APOE and beta-migrating VLDL, and inhibits the binding of beta-VLDL and LDL receptor-related proteins. And inhibition of APOC-1 expression inhibits cell proliferation and induces cell death. |
| NCHL1 | It is a protein that plays a role in extracellular matrix and cell adhesion in the development of neural organs and synaptic plasticity. It promotes soluble and membranous neurite inheritance and inhibits neuronal cell death in cerebellum and hippocampus. It regulates the location and role of connections of pyramidal neurons and the number of neurons and the efficacy of GABA-responsive synapses. It plays a role in regulating cell migration in neuronal regeneration and cortical development, enables integrin-dependent cells to migrate to extracellular matrix proteins, and supplements ANK3 to cell membranes. |
| CAH1 | Catalytic activity: $H_2CO_3 = CO_2 + H_2O$.<br><br>Cofactor : $Zn^{2+}$<br>Enzyme regulation<br>It is activated by histamine, imidazole, L-adrenaline, L- and D-histidine, and L- and D-phenylalanine.<br>It is inhibited by coumarins, sulfonamide derivatives such as acetazolamide, benzene sulfonamide and derivatives (4-carboxyethyl benzene-sulfonamide, 4-carboxyethylbenzene-sulfonamideethyl ester, 4-(acetyl-2-aminoethyl) benzene-sulfonamide, 4-aminoethylbenzene-sulfonamide), and 'prong inhibitors' BR15, BR17, BR22 and BR30 |

| Protein name | Function |
|---|---|
| | Kinetics<br>KM=4.0 mM for CO2<br>KM=15 mM for 4-nitrophenyl acetate<br>CA1 is a potential oncogene and contributes to abnormal cell calcification, apoptosis and migration in breast cancer.<br>It promotes breast calcification by catalyzing the hydration of CO2 |

**Table 2. Function of the three proteins**

However, the primary candidate formula established in the verification phase is not a formula derived from a quantitative method. While it is useful for comparison between groups such as patient group and normal subject (group), if the test is performed individually without comparison between groups, the decision cutoff value may change every time. Therefore, it was necessary to establish an early breast cancer screening model formula (algorithm) by establishing a test method as a quantitative method capable of individual evaluation rather than comparing between groups, and calculating the appropriate decision cutoff value. After improving the analysis method as a quantitative method, the researchers at this institution established a second formula (algorithm) for the early breast cancer screening model suitable for the quantitative method.

The samples used to develop the early breast cancer screening model formula (algorithm) were plasma samples stored in the biorepository of Lab of Breast Cancer Biology (LBCB), the Cancer Research Institute, Seoul National University College of Medicine.

In addition, to prevent bias in the analysis, the order of the samples used for the early breast cancer screening model formula was randomly mixed and then the samples were anonymized by being assigned non-descriptive numbers from 1 to 200. Sample donor information for early breast cancer and normal subjects was blinded to the researchers until analysis was completed. Utilizing the analyzed data, an early breast cancer screening model formula (algorithm) suitable for the quantitative method was established as a second algorithm through logistic regression analysis.

## 1.2. Benefit/Risk Assessment

There will be no direct benefit to each patient for participation in this study. However, in the absence of a hematological marker for diagnosing breast cancer at present, if the 3 biomarkers show a significant difference before/after treatment (depending on the change in the state of cancer), it could be positioned as a new tool that enables early diagnosis of breast cancer in the future.

Since the test is performed through blood collection, it is judged that the specific side effects and risks predicted for the patient will be minimal.

# 2. Study Objectives, Endpoints

## 2.1. Study Objectives

This study aims to quantify the estimated breast cancer biomarker protein discovered in previous studies using the proteomics technique with LC/MS/MS, and evaluate the clinical efficacy (diagnostic ability) of the multi-marker algorithm and decision cutoff value derived from previous studies using a new sample group (patient group without histological diagnosis).

And advancement of previously discovered biomarkers and development of additional biomarkers.

## 2.2. Study Endpoints

It was set based on the same value as the previous study results.

| AUC | Sensitivity (%) | Specificity (%) | Positive predicted value (%) | Negative predicted value (%) | Accuracy (%) |
|---|---|---|---|---|---|
| 0.771 | 67.0 | 82.0 | 78.82 | 71.30 | 74.5 |

**Table 3. Validation baseline used on MASTOCHECK confirmatory study for MFDS approval**

- Area Under the ROC Curve (Concordance index)
- Sensitivity
- Specificity
- Predictive values
- Positive predictive values
- Negative predictive values
- Accuracy

# 3. Stakeholder Information

## 3.1. Name and address of coordinating institution

**Name:** Seoul National University Hospital (SNUH)

- **Address**: 101 Daehak-ro (28 Yeongeon-dong), Jongno-gu, Seoul

## 3.2. Name and position of coordinating investigator and co-investigators

### 3.2.1. Coordinating investigator

Professor Hyeong-Gon Moon, Department of Surgery, Seoul National University Hospital

### 3.2.2. Co-investigators

- Professor Dong-Young Noh, Department of Surgery, Seoul National University College of Medicine, Seoul National University Hospital, Seoul
- Clinical Professor Yumi Kim, Department of Surgery, Seoul National University Hospital, Seoul
- Associate Professor Hee-Chul Shin, Department of Surgery, Seoul National University Bundang Hospital, Seoul, Republic of Korea
- Associate Professor Ki-Tae Hwang, Department of Surgery, Seoul National University College of Medicine, Seoul Metropolitan Government Seoul National University Boramae Medical Center, Seoul
- Associate Professor Junwon Min, Department of Surgery, Dankook University College of Medicine, Cheonan Assistant Professor Soo Kyung Ahn, Breast & Thyroid Center, Department of Surgery, Hallym University, Kangnam Sacred Heart Hospital, Seoul
- Assistant Professor Min Kyoon Kim, Department of Surgery, Chung-Ang University College of Medicine, Seoul Medical Specialist So-Youn Jung, Center for Breast Cancer, National Cancer Center, Goyang
- Assistant Professor Tae-Kyung Yoo, Department of Surgery, Seoul St. Mary's Hospital, College of Medicine, The Catholic University of Korea Professor Hyukjai Shin, Breast and Thyroid Care Center, Myongji Hospital, Gyeonggi-do
- Professor Min Sung Chung, Department of Surgery, Hanyang University College of Medicine, Seoul Professor Sang Uk Woo, Department of surgery, Korea University College of medicine, Seoul
- Associate Professor SeungPil Jung, Division of Breast and Endocrine Surgery, Department of Surgery, Korea University Hospital, Korea University College of Medicine, Seoul
- Associate Professor Ju-Yeon Kim, Department of Surgery, Gyeongsang National University School of Medicine and Gyeongsang National University Hospital, Jinju

### 3.2.3. Main contact at coordinating institution

Clinical Professor Yu-Mi Kim, Department of Surgery, Seoul National University Hospital

### 3.2.4. Investigational device manager

Not applicable for this study

## 3.3. Research sponsor name and address

### 3.3.1. Research sponsor name and address

Bertis Healthcare Inc.

- 5th floor of Pacific Plaza, 9, Gangnam-ro, Giheung-gu, Yongin-si, Gyeonggi-do, Republic of Korea

### 3.3.2. Monitor name and address

Head of R&D Center, Sung-Soo Kim

## 3.4. Research funding party name and address

Bertis Healthcare Inc.

- 5th floor of Pacific Plaza, 9, Gangnam-ro, Giheung-gu, Yongin-si, Gyeonggi-do, Republic of Korea

# 4. Study Population

Prospective approval of protocol deviations to recruitment and enrollment criteria, also known as protocol waivers or exemptions, is not permitted.

## 4.1. Inclusion Criteria

### 4.1.1. Test Group (breast cancer suspected)

- Patients who have a lesion of BI-RADS category of ≥C4b through breast cancer imaging (mammography, ultrasound).
- Women over 18 years old
- Patients with no history of other cancer diagnoses, including previous breast cancer
- Patients voluntarily participating in this clinical study with an informed consent

### 4.1.2. Control Group (healthy expected)

- Patients in BI-RADS category C1-2
- Women over 18 years old
- Patients with no history of other cancer diagnoses, including previous breast cancer
- Patients voluntarily participating in this clinical study with an informed consent

Considering that the purpose of this test method is to help screening for breast cancer, we check the possibility of cancer in patients with suspected cancer. In addition, it allows recruiting additional patients corresponding to C1 and C2 in the BI-RADS category that do not require biopsy and compare the blood test values with the final cancer patient.

## 4.2. Exclusion Criteria

- Patients with recurrent breast cancer
- Patients diagnosed with cancer in other organs within 5 years
- Patients with other cancers confirmed by metastasis test
- Systemic condition that makes it difficult to understand and submit informed consent

## 4.3. Participant Discontinuation/Withdrawal from the Study

1. Study subject who refuses to proceed with further study procedures (withdrawal of consent)

2. If the subject cannot be followed up

3. In case of violation of selection criteria/exclusion criteria

4. When additional study participation is deemed inappropriate by the judgment of the investigator

# 5. Study Intervention(s) and Concomitant Therapy

Protein markers will be analyzed in the collected blood. Not an interventional study.

- Using the LC-MS/MS instrument, commercially available stock solution reagents (DTT, IAA, Urea, Trypsin) are used for protein quantification.

## 5.1. Dosage and Administration

Not applicable (this study does not administer drug/investigational medicinal product)

## 5.2. Concomitant Therapy

Not applicable (this study does not use comparator)

## 5.3. Discontinuation of Study Intervention

Not applicable (not an interventional study)

# 6. Study Assessments and Procedures

## 6.1. Study Assessment

### 6.1.1. Observation items

- Patient's age
- Physical examination (height/weight, body mass index)
- Breast cancer stage (surgical patient-stage based on final pathology result, neoadjuvant chemotherapy patient – clinical stage)
- Surgery method

### 6.1.2. Assessment items

- Imaging examination (mammography, ultrasound)
- Quantitative analysis of Carbonic anhydrase 1, Neural cell adhesion molecule L1-like protein, and Apolipoprotein C-1 from collected blood
- Biopsy: For lesions of BI-RADS ≥C4b in imaging examination, a follow-up biopsy is performed. The results of the biopsy and quantitative analysis of blood proteins will be compared (cancer vs. non-cancer).

## 6.2. Sample Handling Procedures

### 6.2.1. Sample collection

For sample collection, 10 ml of blood should be prospectively collected from the patients who agreed to participation in the study. Sample collection should only be performed if the study obtained IRB approval at the participating institution. Collected sample should be aliquoted into 2 EDTA tubes and 2 SST tubes to account for the possibility of hemolysis (2.5ml for each tube). EDTA tubes should be either slowly inverted back and forth or processed with a roller mixer for 5 minutes to mix the sample with the anticoagulant. EDTA tubes should be kept upright and stored at 4°C until centrifugation. The blood contained in the SST tube should be stored at 4°C until centrifugation. Mixing is not required for SST tubes. The time and date of blood collection must be written on the tubes. All collected samples should be shipped to the analysis room on the same day.

### 6.2.2. Plasma and serum separation condition

In the analysis room, plasma and serum are separated through a refrigerated centrifuge. The conditions are 2,100g (3,000rpm) at 4 °C for 20 minutes. Also, write the centrifugation time so that you can know the refrigerated storage time before separation. Blood collected on the same day should be separated on the same day.

### 6.2.3. Sample preparation

Separated plasma and serum are aliquoted at 4°C, and samples for stock are stored at -80°C. The pre-treatment of the sample for analysis is through protein denaturation and trypsin digestion so that the target protein contained in the plasma/serum sample can be peptidized, and the final analysis is conducted with MRM (Multi-Reaction Monitoring) mode of LC- MS/MS (Tandem mass spectrometry).

### 6.2.4. Target marker concentration analysis

The concentration of target markers contained in human plasma/serum is quantified using an isotope substitution standard form (heavy peptide) having the same sequence.

### 6.2.5. Statistical algorithm analysis

In this analysis, based on the concentrations of multiple markers, an algorithm that calculates the correlation and weight of these markers to derive optimal diagnostic accuracy is applied to determine the diagnostic accuracy.

### 6.2.6. Proteomic profiling

Proteomic profiling of collected blood samples for the advancement of the existing developed markers (to be used in this clinical trial) corresponding to Research Objective 2 and development of additional markers.

# 7. Data Analysis

## 7.1. Medical Device Code Name

- **Classification number**: A24500 (Grade 3)
- **Storage location**: 403, Health Care Innovation Park, Seoul National University Bundang Hospital, 172 Dolmar-ro, Bundang- gu, Seongnam-si, Gyeonggi-do
- **Product name**: MASTOCHECK
- **Product category**: in vitro diagnostic (IVD) software
- **Model name**: BM_1BCM program
- **Ministry of Food and Drug Safety (MFDS) Approval Date**: 03 JAN 19

## 7.2. Data Analysis Procedure

Biomarkers are quantitatively analyzed by the proteomics analysis method for the blood collected through blood collection. The primary breast cancer screening model formula [Result = (0.604 X CAH1 concentration) + (7.7575 X NCHL1 concentration) - (0.523 X APOC1 concentration)] was established by using logistic regression analysis for the 3 proteins (Carbonic anhydrase 1; CAH 1, Neural cell adhesion molecule L1-like protein; NCHL1, Apo lipoprotein C-1; APOC1) finally selected through verification in the previous study. The quantitative analysis results of the obtained samples will be processed by the algorithm and the results will be compared to the results of the previous studies. An independent data management committee will be established to ensure efficiency and objectivity of data analysis and data analysis may include co-investigators and others who are not related to this study.

**<Protein quantitative analysis method>**

① Instruments and tools used for quantitative analysis of 3 types of proteins

Instrument: LC-MS/MS (model name: API 5000, manufacturer: SCIEX, country of manufacture: Singapore, Korean medical device license number: Seoul, No. 10-1245)

Software: AB SCIEX's Analyst 1.6

Reagent: Commercially available stock solution reagent (DTT, IAA, Urea, Trypsin)

② Analyzer conditions

The pretreated plasma sample is expected to be quantitatively analyzed under the following LC-MS/MS conditions.

Analysis method: mass spectrometry (LC-MS/MS)

Detector: API 5000 Mass Spectrometer (Korean medical device license number: Seoul No. 10-1245)

MS/MS condition: Multiple Reaction Monitoring (MRM), Positive mode

# 8. Statistical Considerations

## 8.1. Sample Size Calculation

The researchers at this investigational site requested Seoul National University Hospital Medical Research Cooperation Center (MRCC) to calculate the number of study subjects. The correspondence with MRCC is quoted below:

---

We would like to confirm the diagnostic efficacy of the non-patient group for the breast cancer diagnostic index discovered by proteomics technique.

In order to estimate the sensitivity in the non-patient group, when the sensitivity value is p, the equation for calculating the number of subjects required to ensure their estimation accuracy to w% (half the width of the 95% confidence interval) is as follows:

$$n = \frac{Z_{\alpha/2}^2 \, p(1-p)}{w^2}$$

| alpha/2 | Accurac y | Ratio | Number of subjects | Number of subjects considering the prevalence rate of 60% | Number of subjects considering the 10% dropout rate |
|---|---|---|---|---|---|
| 0.025 | 0.06 | 0.60 | 257 | 429 | 477 |

**Table 4. Study subjects according to accuracy and ratio**

To calculate the number of subjects required for this study, a sensitivity of 60% is assumed by taking into account the sensitivity of 71.58% in the previous study. The number of subjects required to obtain estimation with 60% sensitivity and within 6% of precision is 257 for each group. Assuming the prevalence of 60%, the number of subjects required is calculated as a total of 429 subjects. In addition, if the 10% dropout rate is considered, a total of 477 subjects is required.

*** After recruiting 300 subjects, the total number of patients to be recruited can be recalculated based on the events that occurred up to that point (calculation of the number of N-size by MRCC will be used as the reference).**

| Final Assessment Categories | | | |
|---|---|---|---|
| **Category** | | **Management** | **Likelihood of cancer** |
| 0 | Need additional imaging or prior examinations | Recall for additional imaging and/or await prior examinations | n/a |
| 1 | Negative | Routine screening | Essentially 0% |
| 2 | Benign | Routine screening | Essentially 0% |
| 3 | Probably Benign | Short interval follow-up (6 month) or continued | >0% but ≤2% |
| 4 | Suspicious | Tissue diagnosis | 4a. low suspicion for malignancy (>2% to ≤10%)<br>4b. moderate suspicion for malignancy (>10% to ≤50%)<br>4c. high suspicion for malignancy (>50% to <95%) |
| 5 | Highly suggestive of malignancy | Tissue diagnosis | ≥95% |
| 6 | Known biopsy-proven | Surgical excision when clinically appropriate | n/a |

**Table 5. BI-RADS Assessment Categories**

## 8.2. Randomization method

This study will not require blinding of subjects. Thus, only the investigators will be blinded to the information regarding blood samples. In order to prevent bias that may affect the test results during analysis, only the identification code of the sample will be displayed, and information on each sample will not be disclosed until statistical analysis.

## 8.3. Interim Analysis

Not applicable

# 9. Safety and Adverse Events

The study will be conducted only with collected samples, and there will be no additional invasive testing methods or drug administration for patients. Therefore, it is judged that there will be very few possible adverse effects to the participants (patients).

# 10. Supporting Documentation and Operational Considerations

## 10.1. Regulatory and Ethical Considerations

- This study will be conducted in accordance with the protocol and with the following:
  - Declaration of Helsinki (revised in 2013)
  - Applicable ICH Good Clinical Practice (GCP) guidelines
  - Applicable laws and regulations
- The protocol, protocol amendments, ICF, and other relevant documents must be submitted to an IRB by the investigator and reviewed and approved by the IRB before the study is initiated.
- Any amendments to the protocol will require IRB/IEC approval before implementation of changes made to the study design, except for changes necessary to eliminate an immediate hazard to study participants.

## 10.2. Informed Consent Process

- Investigator who will explain to study subjects and obtain consent: co-investigator of each participating institution
- Person who will provide consent: Study subject
- Duration between the process of explaining the study and the process of obtaining consent: Informed consent is planned to be obtained on the scheduled date of outpatient clinic visit for confirming the result of imaging examination (Informed consent process is expected to take approximately an hour).
- Method to minimize the possibility of coercion: Obtain consent from the patients voluntarily. (Explanation for obtaining consent should be made by the investigator (the principal investigator and co-investigator) specified in the clinical study protocol.
- Language used by the investigator in the process of explaining the research and obtaining consent: study information should be provided in terms that can be understood by the study subject using the study informed consent form
- Informed consent form provided to the study subject or lawfully authorized representative

## 10.3. Compensation plan for study subject

- Compensation provided to study subjects: Financial compensation including round-trip transportation expenses
- After consent is given, the amount of compensation will be disclosed and paid to the subject promptly after the relevant visit (prevention of unfairness such as participation in research due to financial compensation by not mentioning compensation prior to consent)
- The compensation specified above are rewards for the subject consenting to the study and collecting blood. Therefore, it is not contingent on continued study participation. This study is terminated when statistical analysis is performed after blood collection and protein analysis in blood. The duration of the study is also short. Therefore, there is no need for the subject to continue to participate in the study.

## 10.4. Recruitment strategy

Among the patients who visited the investigational sites, patients who satisfy the inclusion criteria for breast imaging examination and able to provide informed consent for study participation after receiving study information from the medical staff (principal investigator or co-investigators) will be recruited as subjects.

## 10.5. Personal information protection plan of study subjects

Patient information in this hospital will be anonymized and managed from the time the sample is collected. Information that can identify individuals such as name and social security number will be redacted from clinical information. Only the analysis results will be shared and the raw data itself will not be disclosed. Information collected for research will be stored in an access-controlled physical location, in a file encrypted with a password. File access rights will be restricted to the principal investigator and co-investigator. When the research results are announced, the individual's identity will be announced in a form that cannot be identified, and case records after the end of the study will be kept separately by the principal investigator until thesis is published and approved, and it will be discarded under the guidance of the principal investigator according to Article 16 of the Enforcement Decree of the Personal Information Protection Act after storage for 3 years after the end of the study.

## 10.6. How to store and dispose of human materials

(The information available on the web portal of the Institutional Bioethics Committee was used as reference)

*1) Purpose of human-derived materials*

After conducting study on the clinical efficacy of the biomarker that has already been developed, the collected blood samples are used to advance the existing biomarker and develop additional biomarkers for the remaining human-derived materials.

*2) Matters concerning the protection and processing of personal information*

Same as the personal information protection method in 9.5.

*3) Matters concerning the preservation and disposal of human-derived materials*

Human-derived materials that have passed the preservation period determined by the research subject after obtaining a written consent for the study of human-derived materials from the study subject will be disposed of in accordance with the standards and methods pursuant to Article 13 of the [Waste Management Act]. When the study is abnormally discontinued, human- derived materials will be transferred according to the procedure prescribed by law.

**< Disposal and transfer recording>**

a) When disposing of human-derived materials according to the standards and methods pursuant to Article 13 of the 「Waste Management Act」, information on disposal such as the date of disposal, amount of disposal, and method of disposal, etc., must be recorded and stored for 5 years from the date of disposal .(Enforcement Regulations Article 36 (2))
b) When transferring human-derived materials to the Human Resources Bank or the Centers for Disease Control and Prevention, transfer the management ledger of human-derived materials in Attachment No. 35 together with matters related to the provision, such as the date and amount of provision, the person who provided, and the person receiving the provision. (Enforcement Rule Article 36 (3))

*4) Matters concerning the provision of human-derived materials and genetic information obtained therefrom*

When a study subject requests disclosure of information about himself or herself, it may be provided in accordance with relevant laws and regulations.

*5) Withdrawal of consent, treatment of human-derived materials upon withdrawal of consent, rights of human-derived material donors, and change of research purpose*

Subjects may decide not to participate in the study at any time and may also discontinue the study. Upon withdrawal of consent, human-derived materials should be disposed of as soon as possible in accordance with relevant laws and regulations. If there is a change in the purpose of the study, the subject is notified again and consent is obtained.

*6) Matters concerning the disposal or transfer of human-derived materials in case of abnormal discontinuation of research on human-derived materials*

It is planned to transfer human-derived materials in accordance with the procedure prescribed by the law.

*7) Matters concerning the retention period and information disclosure of research results of human materials*

**< Records to be kept >**

a) Clinical study protocol and results of deliberation by the institutional committee that deliberated the relevant study (In the case of changes, the revised clinical study protocol and deliberation results are included)
b) Written consent received from a donor of human origin or an approval for exemption from the written consent of the institutional committee pursuant to Paragraph 3 of the same Article
c) Collection, use and provision of personal information
d) Research end report including research results, and the results of investigation and supervision of the institutional committee on the progress and results of the research pursuant to Article 10 (3) 2 of the Act

**< Storage period >**

The investigator will keep the above records for 3 years from the time the study is completed.

**< Record destruction >**

Among documents that have passed the storage period, matters related to personal information will be destroyed in accordance with Article 16 of the Enforcement Decree of the Personal Information Protection Act. However, if storage is necessary for subsequent research or record accumulation, the storage period may be extended after deliberation by the institutional committee of the affiliated institution.

**< Disclosure >**

a) A donor of human-derived material (hereinafter referred to as “claimer” including a legal representative) may request disclosure of information about himself or herself.
b) The investigator who received the request shall disclose the information requested for disclosure unless there is a special reason.
c) All information disclosure procedures will be carried out through the institutional committee in accordance with Article 16 of the Enforcement Rule of the Bioethics Act.
d) The investigator must submit the information requested by the applicant to the institutional committee within 30 days from the date of receiving the request for disclosure of form information in Attachment 7 submitted by the applicant from the institutional committee.
e) However, if information cannot be disclosed due to a special reason, the reason must be submitted to the institutional committee.

# 11. References

1. R. Tuli,R.A. Flynn, K.L. Brill et.al. Diagnosis, Treatment, and Management of Breast Cancer in Previously Augmented Women. Breast J. 12(4), 343-348, 2006

2. Z. Rui, J. Jian-Guo, T. Yuan-Penget al. Use of Serological Proteomic Methods to Find Biomarkers Associated With Breast Cancer. Proteomics. 3(4), 433-439. 2003.

3. Gromov P, Gromova I. et. al. Up-regulated proteins in the fluid bathing the tumour cell microenvironment as potential serological markers for early detection of cancer of the breast. Mol Oncol, 4: 65-89, 2010.

4. Shi Q, Harris L.N. et. al.Declining plasma fibrinogen alpha fragment identifies HER2-positive breast cancer patients and reverts to normal levels after surgery. J Proteome Res, 5: 2947-2955, 2006.

5. BI-RADS Tutorial by G. Pfarl, MD & T.H. Helbich, MD, Department of Radiology, University of Vienna.

6. Chang J.W, Kang U.B. et. al. Identification of circulating endorepellin LG3 fragment: Potential use as a serological biomarker for breast cancer. Proteomics Clin Appl, 2: 23-32, 2008.

7. Shi Q, Harris L.N. et. al. Declining plasma fibrinogen alpha fragment identifies HER2-positive breast cancer patients and reverts to normal levels after surgery. J Proteome Res, 5: 2947-2955, 2006.

8. Fan Y, Wang J. et. al. Detection and identification of potential biomarkers of breast cancer. J Cancer Res Clin Oncol, 136: 1243-1254, 2010.

9. Gromov P, Gromova I. et. al. Up-regulated proteins in the fluid bathing the tumour cell microenvironment as potential serological markers for early detection of cancer of the breast. Mol Oncol, 4: 65-89, 2010.

10. Kang U.B, Ahn Y. et. al. Differential profiling of breast cancer plasma proteome by isotope-coded affinity tagging method reveals biotinidase as a breast cancer biomarker. BMC Cancer, 10: 114, 2010.

11. Lee H.B., Kang U.B., Moon H.G. et al. Development and Validation of a Novel Plasma Protein Signature for Breast Cancer Diagnosis by Using Multiple Reaction Monitoring-based Mass Spectrometry. Anticancer Res. 35(11), 6271-6279, 2015
