# Supplementary material for: Diagnostic accuracy of a three-protein signature in women with suspicious breast lesions: a multicenter prospective trial
Source: Breast Cancer Res. 2023 Feb 14;25:20. doi: 10.1186/s13058-023-01616-5 (PMC9930228; doi:10.1186/s13058-023-01616-5)
Supplement: Supplementary file 2 — Additional file 2. Additional Methods. [file 13058_2023_1616_MOESM2_ESM.docx]

**Additional Methods**

**Further information regarding study design**

The Institutional Review Board (IRB) of Seoul National University Hospital (SNUH) recommended a blinded analysis of the trial data by the SNUH Medical Research Collaboration Center (MRCC) due to the conflicts of interest of several participating investigators. These are listed in the Conflict of Interest section. The data presented here are the results of the analysis performed by the MRCC without the investigators’ participation. All statistical analyses were done with SAS version 9.4 (SAS institute, Cary, NC, USA). IRB or independent ethics committee approval was obtained from each participating site, and the study was conducted in accordance with the principles of the Declaration of Helsinki.

**Sample collection and processing**

Blood samples were collected with ethylenediaminetetraacetic acid (EDTA) tubes and serum separator tubes (Vacutainer, Becton Dickinson, Franklin Kakes, NJ, USA) from 13 participating hospitals and transferred to the central laboratory (Bertis Research Institute, Korea). Samples were centrifuged at 2,100 × g for 20 min, aliquoted, and stored at -80 °C until further processing. Samples with signs of overt hemolysis were excluded from the study.

Protein denaturation and digestion were performed as previously described (1). First, 2.0 µl of plasma was denatured in 8 M urea and reduced with 10 mM of dithiothreitol (DTT) and 100 mM of ammonium bicarbonate (pH 7.8) for 1.5 hours at 35 °C. The samples were alkylated with 20 mM iodoacetamide for 30 min in the dark at room temperature. The urea concentration was reduced to 1 M with 100 mM ammonium bicarbonate prior to digestion to avoid interference by urea from protein digestion. Protein digestion was performed with 2.0 µg of sequencing-grade trypsin (Promega, Fitchburg, WI, USA) at 37 °C for 16 hours. The reaction was terminated with the addition of trifluoroacetic acid (TFA) to a final concentration of 1.0%. Three synthetic isotopically labeled peptides (SIS-labeled peptides, target surrogate peptides: APOC1, CA1, and CHL1) were spiked into the trypsin-digested sample, which was then desalted and concentrated by solid phase extraction on a Sepak cartridge (100 mg, Waters, Milford, MA, USA). The eluted samples were lyophilized by vacuum centrifugation and stored at -80°C prior to MS. MRM-MS analysis was performed on a Qtrap 5500 system (AB Sciex, Framingham, MA, USA). The lyophilized samples were reconstituted in 0.1% formic acid. Samples were injected in a 5 µl volume in a C18 reverse-phase column at a flow rate of 10 µl/min. For the calibration of MS, linear dilution series of the SIS-peptide mixtures were spiked into the samples at six concentration levels of the SIS-labeled peptides. The area under the highest intensity peak was calculated using the Multiquant (version 3.0.3, AB Sciex) software to quantify the target markers. The value calculated from the area under the curve was fitted to a standard curve to obtain the quantification value. The results of the three-protein signature were determined using a regression algorithm, as approved by the Korean Ministry of Food and Drug Safety. Briefly, the concentration values (ng/ml) were used as the input data for the algorithm. The concentration of each protein was calculated using the intensity ratio between internal standards (stable isotope heavy peptide standards with constant concentration) and the quantified marker’s intensity. The cut-off value was set to maximize sensitivity and specificity (1). If the result was equal to or greater than the cut-off value, it indicated that the lesion is highly suspicious for breast malignancy, and a result under the cut-off value suggested low suspicion.

**Statistical design and analysis**

The primary outcome was the sensitivity of the three-protein signature to detect breast malignancies. The sample size was calculated according to the hypothesis that the three-protein signature would show sensitivity over 60% with 0.06 precision of the estimate. We assumed that breast malignancy prevalence among women with moderate-to-highly suspicious lesions based on BI-RADS classification (4B, 4C, 5) was approximately 60% based on our experience and a previous report (2). Based on this prevalence, we calculated 257 confirmed cases of breast malignancy would be needed; therefore, 429 women would need to be enrolled.

**Supplementary references**

1. Kim Y, Kang UB, Kim S, Lee HB, Moon HG, Han W, et al. A validation study of a multiple reaction monitoring-based proteomic assay to diagnose breast cancer. J Breast Cancer. 2019;22(4):579-86.

2. Elverici E, Barca AN, Aktas H, Ozsoy A, Zengin B, Cavusoglu M, et al. Nonpalpable BI-RADS 4 breast lesions: sonographic findings and pathology correlation. Diagn Interv Radiol. 2015;21(3):189-94.
