# Supplementary material for: Diagnostic accuracy of a three-protein signature in women with suspicious breast lesions: a multicenter prospective trial
Source: Breast Cancer Res. 2023 Feb 14;25:20. doi: 10.1186/s13058-023-01616-5 (PMC9930228; doi:10.1186/s13058-023-01616-5)
Supplement: Supplementary file 3 — Additional file 3. Additional Table 1 and Additional Table 2. [file 13058_2023_1616_MOESM3_ESM.docx]

**Additional Table 1. All participants’ demographic and clinicopathological information**

| **Variables** |  | **Number of women (%)** | **Remarks** |
| --- | --- | --- | --- |
|  |  | **All participants**  **(n=642)** |  |
| Age | Mean±SD | 52.2±11.6 |  |
| Height (cm) | n | 549 (85.5%) |  |
|  | Unknown | 93 (14.5%) |  |
|  | Mean±SD | 158.4±5.9 |  |
| Weight (kg) | n | 549 (85.5%) |  |
|  | Unknown | 93 (14.5%) |  |
|  | Mean±SD | 59.3±9.6 |  |
| All medication history | No | 403 (62.8%) |  |
|  | Yes | 239 (37.2%) |  |
| Breast density grade in mammogram^a^ | n | 269 (41.9%) |  |
|  | Unknown | 373 (58.1%) |  |
| BI-RADS category | Category 1 and 2 | 191 (29.8%) |  |
|  | Category 4B | 223 (34.7%) |  |
|  | Category 4C | 84 (13.1%) |  |
|  | Category 5 | 144 (22.4%) |  |
| Three-protein signature results (Plasma) | High suspicion for breast malignancy | 342 (53.3%) | Concordant in 605 (94.1%) women |
|  | Low suspicion for breast malignancy | 300 (46.7%) |  |
| Three-protein signature results (Serum) | High suspicion for breast malignancy | 331 (51.6%) |  |
|  | Low suspicion for breast malignancy | 311 (48.4%) |  |
|  |  | **Women with breast malignancy**  **(n=313)** |  |
| Histologic type | Invasive ductal carcinoma | 244 (78.0%) |  |
|  | Invasive lobular carcinoma | 22 (7.0%) |  |
|  | Others | 47 (15.0%) |  |
| AJCC 7^th^ staging system | Unknown | 61 (19.5%) |  |
|  | 0 | 29 (9.3%) |  |
|  | 1 | 101 (32.3%) |  |
|  | 2 | 90 (28.8%) |  |
|  | 3 | 28 (8.9%) |  |
|  | 4 | 4 (1.3%) |  |
| Breast cancer subtypes | Unknown | 61 (19.5%) |  |
|  | HR+/HER2- | 172 (55.0%) |  |
|  | HR+/HER2- | 18 (5.8%) |  |
|  | HR+/HER2- | 36 (11.5%) |  |
|  | HR-/HER2- | 26 (8.3%) |  |

*Abbreviation:* SD; standard deviation, BI-RADS; Breast Imaging Reporting And Data System, AJCC; American Joint Committee on Cancer, HR; Hormone Receptor, HER2; human epidermal growth factor receptor 2

^a^American College of Radiology (ACR) BI-RADS Breast Density Categories

**Additional Table 2. Demographic characteristics according to the classification of participants**

| **Variables** |  | **Number of women (%)** | | |  |
| --- | --- | --- | --- | --- | --- |
|  |  | **Participants with breast malignancy (n=313)** | **Participants with benign breast lesion**  **(n=138)** | **Participants without suspicious breast lesion**  **(n=191)** | ***p-value*** |
| Age | Mean±SD | 56.1±10.6 | 47.2±10.9 | 49.4±10.8 | *<0.001^1)^* |
| Height (cm) | n | 287 (91.7%) | 120 (87%) | 142 (74.3%) | *<0.001^1)^* |
|  | Unknown | 26 (8.3%) | 18 (13%) | 49 (25.7%) |  |
|  | Mean±SD | 157.3±6.1 | 159.8±5.9 | 159.2±4.9 |  |
| Weight (kg) | n | 287 (91.7%) | 120 (87.0%) | 142 (74.3%) | *0.619^1)^* |
|  | Unknown | 26 (8.3%) | 18 (13.0%) | 49 (25.7%) |  |
|  | Mean±SD | 58.7±10.0 | 58.6±9.8 | 58.4±8.5 |  |
| Anti-hypertensive medication | No | 241 (77.0%) | 119 (86.2%) | 170 (89.0%) | *0.601^2)^* |
|  | Yes | 72 (23.0%) | 19 (13.8%) | 21 (11.0%) |  |
| Dyslipidemia medication | No | 262 (83.7%) | 131 (94.9%) | 167 (87.4%) | *0.004^2)^* |
|  | Yes | 51 (16.3%) | 7 (5.1%) | 24 (12.6%) |  |
| Diabetes medication | No | 284 (90.7%) | 126 (91.3%) | 185 (96.9%) | *0.030^2)^* |
|  | Yes | 29 (9.3%) | 12 (8.7%) | 6 (3.1%) |  |
| Synthroid medication | No | 289 (92.3%) | 133 (96.4%) | 178 (93.2%) | *0.273^2)^* |
|  | Yes | 24 (7.7%) | 5 (3.6%) | 13 (6.8%) |  |
| Other medications | No | 266 (85.0%) | 117 (84.8%) | 168 (88.0%) | *0.601^2)^* |
|  | Yes | 47 (15.0%) | 21 (15.2%) | 23 (12.0%) |  |

*Abbreviation*: SD; standard deviation

1)Kruskal-Wallis test 2) Chi-square test
